# Book Reviews

**Published:** 1913-04

**Authors:** 


### BOOK REVIEWS

MEN, MEDICINE AND MANNERS. Octavo, uncut, in heavy paper cover. Price postpaid, One Dollar. By Medicus Peregrinus. W. M. Leonard, Publisher, Boston, Mass.

The Essays and Sketches which make up this collection originally appeared from time to time in the columns of the Boston Medical and Surgical Journal. They represent the observations of a doctor, from his professionl point of view, on men and books and other phenomena, especially in relation to medicine. The reader may be not only entertained but instructed, as he realizes how abundantly the doctor's life affords special opportunities for contact with larger interests outside the day's work.

---

THE OPERATING ROOM AND THE PATIENT. By Russell S. Fowler, M. D., Chief Surgeon First Division, German Hospital, Brooklyn, New York. Third Edition Rewritten and Enlarged Octavo volume of 611 pages with 212 illustrations. Philadelphia and London: W. B. Saunders Company, 1913. Cloth, $3.50 net.

Every house surgeon should be required to read this book every six months during his hospital service. Two important things would result, namely: Many serious calamities would be obviated and a much better surgeon would enter practice at the end of his service. The book is most practical and useful.

GOLDEN RULES OF GYNECOLOGY. By George B. Norberg, M. D., Professor of Diseases of Women and Clinnical Gynecology, University Medical College, Kansas City, Mo., Gynecologist to Kansas City General Hospital, Fellow and Ex-President Kansas City Academy of Medicine. 250 pages. 8 vo. Price $2.25 C.V. Mosey & Co., St. Louis, Mo.

There is need for just such a book as this one. It does not displace the text-book or the monograph on gynecology, but is rather a guide to what one should know and observe on this fascinating branch of medicine.

In 250 pages one finds the really "Golden Rules," the observance and application of which will enable the practitioner of medicine to get results. Convenient in size and convincingly written, this volume can be perused over and over again with the feeling that each time it is read one becomes better able to cope with diseases of women.

---

EPIDEMIC CEREBROSPINAL MENINGITIS. By Albraham Sophin, M. D., formerly with the New York Research Laboratory. Twenty-three Illustrations. Pp 272. Published by C V. Mosby Co., St. Louis, Mo.

Sophian has utilized his rare opportunity in studying this subject in the Research Laboratory of New York as well as in the Texas Epidemic and in his work conveys a thorough yet simple description of the diagnosis and treatment of cerebrospinal meningitis—a disease now demanding especial attention.

---

OPHTHALMOLOGY FOR VETERINARIANS. By Walter N. Sharp, M. D., Professor of Ophtalmology in the Indiana Veterinary College 12mo of 210 pages, illustrated. Philadelphia and London: W. B. Saunders Company, 1913. Cloth, $2.00 net.

This monograph covers well and concisely this important subject in veterinary surgery. The literature on diseases of the eye is so limited it should fulfill a well defined want.

THE SURGICAL CLINICS OF JOHN B. MURPHY, M. D., at Mercy Hospital, Chicago. Volume I. Number VI. (December). Octavo of 153 pages, illustrated. Philadelphia and London: W. B. Saunders Company, 1912. Published Bi-Monthly. Price per year: Paper, $8.00. Cloth, $12.00.

The subjects discussed in this number are: Carcinoma of the breast; treatment of malignant tumors with rochoactive substances; solpingitis-pelvic infection; metastatic gonorrhoeal arthritis of the knee; ankylosis of the elbow—arthroplasty; fracture of the patella; ununited fiacture of the femur; etc.

